# Enhancement effect of mass imbalance on Fulde-Ferrell-Larkin-Ovchinnikov type of pairing in Fermi-Fermi mixtures of ultracold quantum gases

**DOI:** 10.1038/srep39783

**Published:** 2017-01-04

**Authors:** Jibiao Wang, Yanming Che, Leifeng Zhang, Qijin Chen

**Affiliations:** 1 Department of Physics and Zhejiang Institute of Modern Physics, Zhejiang University, Hangzhou, Zhejiang 310027, China; 2 Synergetic Innovation Center of Quantum Information and Quantum Physics, Hefei, Anhui 230026, China

## Abstract

Ultracold two-component Fermi gases with a tunable population imbalance have provided an excellent opportunity for studying the exotic Fulde-Ferrell-Larkin-Ovchinnikov (FFLO) states, which have been of great interest in condensed matter physics. However, the FFLO states have not been observed experimentally in Fermi gases in three dimensions (3D), possibly due to their small phase space volume and extremely low temperature required for an equal-mass Fermi gas. Here we explore possible effects of mass imbalance, mainly in a ^6^Li–^40^K mixture, on the one-plane-wave FFLO phases for a 3D homogeneous case at the mean-field level. We present various phase diagrams related to the FFLO states at both zero and finite temperatures, throughout the BCS-BEC crossover, and show that a large mass ratio may enhance substantially FFLO type of pairing.

The past decade has seen great progress in ultracold atomic Fermi gas studies^1^,^2^. With the easy tunability in terms of interaction, dimensionality, population imbalance as well as mass imbalance^1^,^2^, ultracold Fermi gases have provided a good opportunity to study many exotic quantum phenomena. In particular, the Fulde-Ferrell-Larkin-Ovchinnikov (FFLO) states, which were first predicted by Fulde and Ferrell^3^ (FF) and Larkin and Ovchinnikov^4^ (LO) in an *s*-wave superconductor in the presence of a Zeeman field about fifty years ago, have attracted enormous attention in condensed matter physics^5^, including heavy-fermion^6^, organic^7^ and high *T*_*c*_ superconductors^8^,^9^,^10^, nuclear matter^11^ and color superconductivity^12^, and ultracold Fermi gases^13^,^14^,^15^. In these exotic states, Cooper pairs condense at a finite momentum **q**, with an order parameter of the form of either a plane-wave Δ(**r**) = Δ_0_*e*^*i***q** ⋅ **r**^ or a standing wave 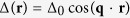 for the FF and LO states, respectively. Despite many theoretical studies on the FFLO states in equal-mass Fermi gases, both in a 3D homogeneous case^16^,^17^,^18^,^19^,^20^,^21^ and in a trap^22^,^23^,^24^, the experimental search for these exotic states in atomic Fermi gases still has not been successful^25^,^26^,^27^, largely because they exist only in a *small* region at *very low* temperature in the phase space^16^,^17^,^18^,^23^. To find these elusive states, attention has been paid to more complex systems. There have been theoretical investigations in either Fermi-Fermi mixtures^28^,^29^,^30^ or equal-mass Fermi gases with spin-orbit coupling^31^,^32^,^33^,^34^,^35^,^36^,^37^ or in an optical lattice^38^,^39^,^40^,^41^,^42^,^43^. Recently, Stoof and coworkers^28^,^29^ found an instability toward a supersolid state (i.e., the LO state) in a homogeneous ^6^Li–^40^K mixture in the unitarity and BCS regimes. Using a mean-field theory and the Bogoliubov–de Gennes (BdG) formalism, they have also studied the LO states for the unitary case^30^. However, it is hard to perform stability analysis for various phases in the BdG formalism. Other types of mass-imbalanced systems such as the ^6^Li-^173^Yb mixture were not considered in ref. 30.

In this paper, we will investigate the *one-plane-wave* FFLO states (i.e., the FF states) in a homogeneous ^6^Li–^40^K mixture, as well as for other mass ratios, as they undergo BCS–BEC crossover, using a mean-field theory. In particular, we will focus on the effect of a varying mass ratio. We will present several *T*–*p* (where *p* is the population imbalance) phase diagrams to show the FFLO regions under typical interaction strengths (1/*k*_*F*_*a*) at finite temperature, as well as *p*–1/*k*_*F*_*a* phase diagrams at zero temperature. We find that when the heavy species, ^40^K, is the majority, an *s*-wave FFLO phase, which is stable against phase separation, persists throughout the BCS through BEC regimes, with the population imbalance evolving from small to large. In contrast, when the light species, ^6^Li, is the majority, such an FFLO phase exists only in the BCS regime. At unitarity, the phase space of FFLO states becomes substantially enlarged as the mass ratio increases. The superfluid transition temperature *T*_*c*_ of the FFLO states may be enhanced by a factor of about 3 and 7 for a large mass ratio as in ^6^Li–^40^K^44^,^45^,^46^ and ^6^Li–^173^Yb^47^,^48^, respectively, in comparison with the equal-mass case^16^, which is hardly accessible experimentally^26^. Therefore, one may find it realistic to experimentally observe the exotic FFLO states in unitary ultracold Fermi-Fermi mixtures with a large mass ratio.

We also find that at zero temperature the phase space of stable FFLO states becomes larger as the mass ratio increases. This has never been reported by other previous works.

Despite the fact that a mean-field theory usually overestimates *T*_*c*_, we believe that our findings about the enhancement effect of a large mass ratio on FFLO type of pairing remain valid.

## Theoretical Formalism

We consider a three-dimensional (3D) Fermi-Fermi mixture with a short-range contact potential of strength *U* < 0, where momentum **k** pairs with **q** − **k** and thus Cooper pairs have a nonzero center-of-mass momentum **q**. The dispersion of free atoms is given by **ξ**_**k**,*σ*_ = **k**^2^/2*m*_*σ*_ − *μ*_*σ*_, where *m*_*σ*_ and *μ*_*σ*_ are the mass and chemical potential for (pseudo)spin *σ* = ↑, ↓, respectively. We set the volume *V* = 1, and 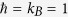. At the mean-field level, the system with a one-plane wave LOFF solution can be described by the following Hamiltonian


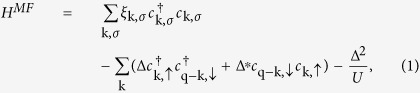


where the order parameter Δ carries momentum **q**. Using Bogoliubov transformation, it is easy to deduce the gap equation via the self-consistency condition


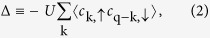


which can be written as


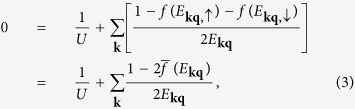


where 
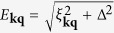
, *E*_**kq**,↑_ = *E*_**kq**_ + *ζ*_**kq**_, *E*_**kq**,↓_ = *E*_**kq**_ − *ζ*_**kq**_, *ξ*_**kq**_ = (*ξ*_**k**,↑_ + *ξ*_**q−k**,↓_)/2, *ζ*_**kq**_ = (*ξ*_**k**,↑_ − *ξ*_**q−k**,↓_)/2. We have defined the average


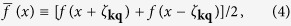


where *f*(*x*) is the Fermi distribution function. The coupling constant *U* can be replaced by the dimensionless parameter, 1/*k*_*F*_*a*, via the Lippmann-Schwinger equation


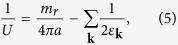


where *a* is the *s*-wave scattering length, *m*_*r*_ = 2*m*_↑_*m*_↓_/(*m*_↑_ + *m*_↓_) is twice the reduced mass, and *ε*_**k**_ = *k*^2^/2*m*_*r*_. Therefore the gap equation becomes


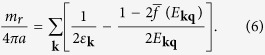


The number density of each species is given by


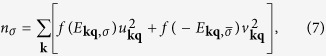


where 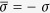, and the coherence factors 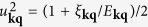, 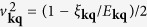. So the total number density *n* = *n*_↑_ + *n*_↓_ and the density difference *δn* ≡ *n*_↑_ − *n*_↓_ are given by


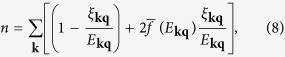



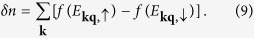


The thermodynamic potential Ω_*S*_ is given by


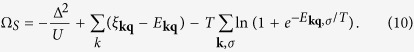


Momentum **q** is determined by minimizing Ω_*S*_ at **q**, i.e., (∂Ω_*S*_)/(∂**q**) = 0, which leads to


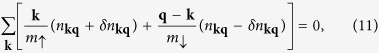


where *n*_**kq**_ and *δn*_**kq**_ are given by the summands of Eqs (8) and (9), respectively. Furthermore, the FFLO solutions are subject to the stability condition against phase separation (PS)^16^,^49^,^50^,


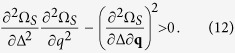


This condition is equivalent to the positive definiteness of the particle number susceptibility matrix {∂*n*_*σ*_/∂*μ*_*σ*′_}^16^,^50^. For the Sarma phase (where *q* = 0), Eq. (12) is reduced to ∂^2^Ω_*S*_/∂Δ^2^0^50^.

Equations (6), (8), (9) and (11) form a closed set of self-consistent equations, which can be used to solve for (Δ, *μ*_↑_, *μ*_↓_, **q**) with various parameters 1/*k*_*F*_*a, p*, and *T*, as well as the mass ratio *m*_↑_/*m*_↓_, and obtain the FFLO regions in phase diagrams. Since phase separation provides an alternative way to accommodate the excessive majority fermions, some of the mean-field solutions of the FFLO states are unstable against phase separation. Here we use the stability condition Eq. (12) to locate the phase boundary separating stable FFLO (or Sarma superfluid) phases and the PS phases. As a convention, we take the heavy (light) species to be spin up (down), and define Fermi momentum *k*_*F*_ = (3*π*^2^*n*)^1/3^. To avoid an artificial jump across population imbalance *p* ≡ *δn*/*n* = 0 in the phase diagrams, we take *m* = (*m*_↑_ + *m*_↓_)/2 and define the Fermi temperature as 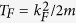 as our energy unit.

Note that for the (**q** = 0) Sarma phases, we will use the pairing fluctuation theory described in ref. 51 to determine the superfluid and pseudogap regions.

## Numerical Results and Discussions

**Figure 1:**
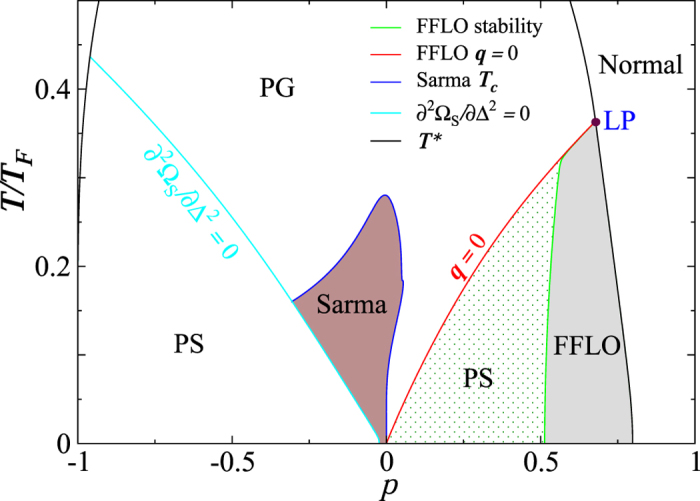
*T*–*p* phase diagram of a homogeneous ^6^Li–^40^K mixture at unitarity. Here “PG” and “PS” indicate pseudogapped normal state and phase separation, respectively, and LP labels a Lifshitz point. An FFLO superfluid (gray shaded) phase exists in the high *p* regime when ^40^K dominates, while it becomes unstable in the dotted region. A Sarma superfluid lives in the intermediate *T* and low *p* regime (brown shaded region).

Figure 1 shows the calculated *T*–*p* phase diagram for a homogeneous ^6^Li–^40^K mixture at unitarity. Pairing takes place below the pairing temperature T^*^ (black solid curve). A mean-field FFLO solution exists to the lower right of the (red) **q** = 0 line. However, stable FFLO states exist only when ^40^K is the majority at relatively high *p* (in the gray shaded area). For lower *p*, FFLO states become unstable and phase separation takes place at low *T* (dotted region), whereas Sarma superfluid (brown area) and pseudogap states exist at intermediate *T*. The (green) line that separates the PS and the FFLO phases is given by the stability condition Eq. (12). When ^6^Li is the majority, i.e., *p* < 0, phase separation dominates the low *T* region. Note here that, as we focus on the FFLO phases, we do not distinguish superfluid and pseudogap states in the PS regions. Further details regarding non-FFLO related phases can be found in ref. 51.

**Figure 2:**
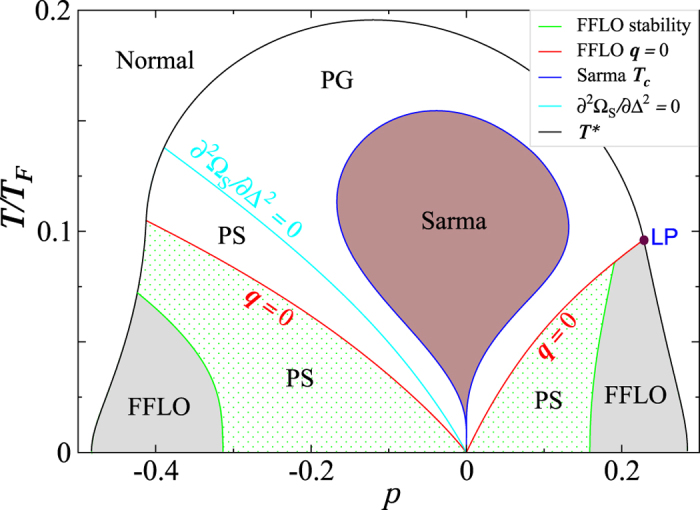
*T*–*p* phase diagram of a homogeneous ^6^Li–^40^K mixture at 1/*k*_*F*_*a* = −1, similar to Fig. 1. Here the FFLO phase (gray shaded regions) exists for both *p* > 0 and *p* < 0.

Shown in Fig. 2 is the (near-)BCS counterpart of Fig. 1 at 1/*k*_*F*_*a* = −1, with much weaker pseudogap effects. Here we find stable FFLO phases for *p* < 0 as well, when ^6^Li is the majority. This is different from refs 28, 29 and 30, which found no LO or supersolid states in the BCS regime for *p* < 0. The *p* > 0 part is rather similar to the unitary case, except that everything moves to lower *p* and lower *T* due to weaker pairing strength. For *p* < 0, the **q** = 0 line splits the PS phase into two regions, representing unstable Sarma (upper) and FFLO (lower part) phases, respectively.

In both Figs 1 and 2, we have found a Lifshitz point (as labeled “LP”) within the mean-field treatment, below which FFLO states emerge.

As the pairing strength grows, the Sarma phase becomes stabilized in a much larger region, especially for *p* < 0 (not shown). However, the stable FFLO states are squeezed towards very low *T* and very high 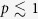, and eventually disappear on the BEC side of the Feshbach resonance. Our result suggests that it is more promising to find FFLO phases in the unitary regime.

**Figure 3:**
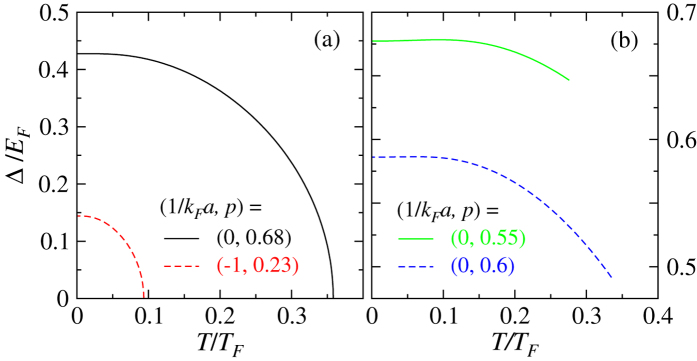
Typical behavior of the order parameter amplitude Δ in the FFLO phase as a function of *T*, for a homogeneous ^6^Li–^40^K mixture. Plotted in (**a**) are the cases at (1/*k*_*F*_*a, p*) = (0,0.68) (black solid) and (−1, 0.23) (red dashed), and in (**b**) are at unitarity with *p* = 0.55 (green solid) and 0.6 (blue dashed line).

Typical behaviors of the order parameter amplitude Δ are shown in Fig. 3 as a function of *T*. Plotted in Fig. 3(a) two cases corresponding to the FFLO phases in Figs 1 and 2, respectively. For both cases, we have chosen a population imbalance such that the FFLO phase turns into a normal gas upon crossing the phase boundary with increasing *T*. The ratio 2Δ(0)/*T*_c_ = 2.39 and 3.08 for the unitary and BCS cases, respectively, substantially lower than the BCS ratio 3.52 for a balanced Fermi gas with equal masses. Its value depends on multiple parameters and can vary substantially as a function of *p*, as shown in the Supplementary Fig. S6.

At the mean-field level, the PG phase would be a Sarma superfluid state. Across the *q* = 0 boundary between this phase and FFLO phase, the transition is of the second order, with a finite but continuous gap across the boundary. This corresponds to the *p* = 0.6 case (blue dashed line) in Fig. 3(b). On the other hand, across the boundary between the PS and FFLO phases, such as the *p* = 0.55 case (green solid line) shown in Fig. 3(b), the transition is of the first order, with a finite but different gaps on the two sides. At fixed *T*, the gap decreases monotonically from the PS-FFLO boundary on the smaller |*p*| side towards the FFLO-normal phase boundary on the larger |*p*| side, as shown in Supplementary Fig. S5.

The curves in Fig. 3(b) reveals that the gap exhibits a weak non-monotonic behavior at low *T*. This is not unusual for population imbalanced Fermi gases, and has been found previously with equal mass yet population imbalanced Fermi gases as well^52^. The reason is that thermal smearing of the Fermi surfaces at finite *T* may alleviate the mismatch between them, and thus enhance pairing in comparison with zero *T*. This is the same physics behind intermediate temperature superfluidity^52^.

**Figure 4:**
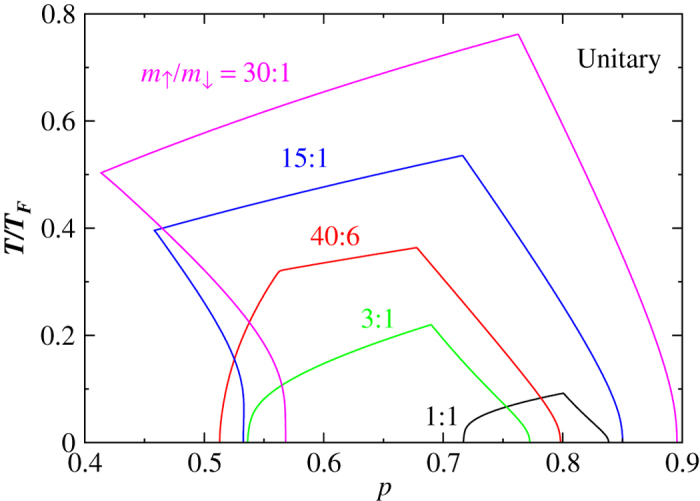
*T*–*p* phase diagram of stable FFLO superfluid in Fermi-Fermi mixtures with different mass ratios (as labeled) at unitarity. Large mass ratio enhances FFLO type of pairing.

To ascertain the effect of a varying mass ratio *m*_↑_/*m*_↓_, we now focus on the stable FFLO superfluid phase for *p* > 0 at unitarity and compute the phase diagram for a series of different mass ratios, as shown in Fig. 4. (The stable FFLO phase for *p* < 0 quickly disappears when 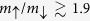). Now that the mass ratio is changing, it is important to pick the right energy unit for meaningful comparison. In addition to the *T*_*F*_ used here, one may alternatively consider using *m*_*r*_ in the definition of *T*_*F*_. The plot is shown in the Supplemental Fig. S2. However, since *m*_*r*_ is an average based on the inverse mass, it puts more weight on the light species, which is more appropriate for the *p* < 0 case. For the large *p* > 0 case, where the heavy species dominates, we conclude that the present definition of *T*_*F*_ is more appropriate.

Figure 4 suggests that the FFLO *T*_*c*_ increases as the mass imbalance grows. At the same time, the phase space of stable FFLO superfluid also grows much larger as the mass ratio increases. In comparison with the mass balanced case, i.e., *m*_↑_/*m*_↓_ = 1, for *m*_↑_/*m*_↓_ = 40:6, the enhancement of the FFLO *T*_*c*_ is about 3 times. For the ^6^Li–^173^Yb mixture^47^,^48^ which has a mass ratio near 30, the enhancement is about 7 times. Such great enhancement of *T*_*c*_ and enlarged phase space suggest that it is much easier to find experimentally the exotic FFLO superfluid with a large mass ratio. Note that one may also consider measuring *T*_*c*_ in units of the actual Fermi temperature of the heavy majority atoms, 
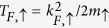
, which seems to be a natural choice for ref. 27. In this case, the enhancement of *T*_*c*_ by mass imbalance would be even more dramatic, being 7 and 16 times, respectively, as shown in the Supplemental Fig. S1.

One may also notice that there is a curvature change in the FFLO-PS phase boundary in Fig. 4, (as well as Supplemental Figs S1 and S2), as the mass ratio increases. For a small mass ratio, the FFLO phase only occupies a small high *p* and low *T* region. As the mass ratio increases, it starts to extend towards the PS phase. This is due to the energetic competition between the FFLO and PS phases at low *T*. The figure suggests that at intermediate *T*, the FFLO phase can extend farther towards low *p* than at lower *T*, when the mass ratio becomes large. It also implies that, when compared with the homogeneous mean-field Sarma state, the energy of the PS phase decreases faster with decreasing *T* than the FFLO case, at an intermediate population imbalance and with a very large mass ratio. In this case, phase separation becomes energetically more favorable and thus the low *T* part of the phase boundary bends towards the FFLO phase. As a consequence, there exists an optimal value of the mass ratio, for which one can reach the FFLO phase with a lowest possible *p*. This observation would be meaningful, if one could continuously tune the mass ratio in experiment.

**Figure 5:**
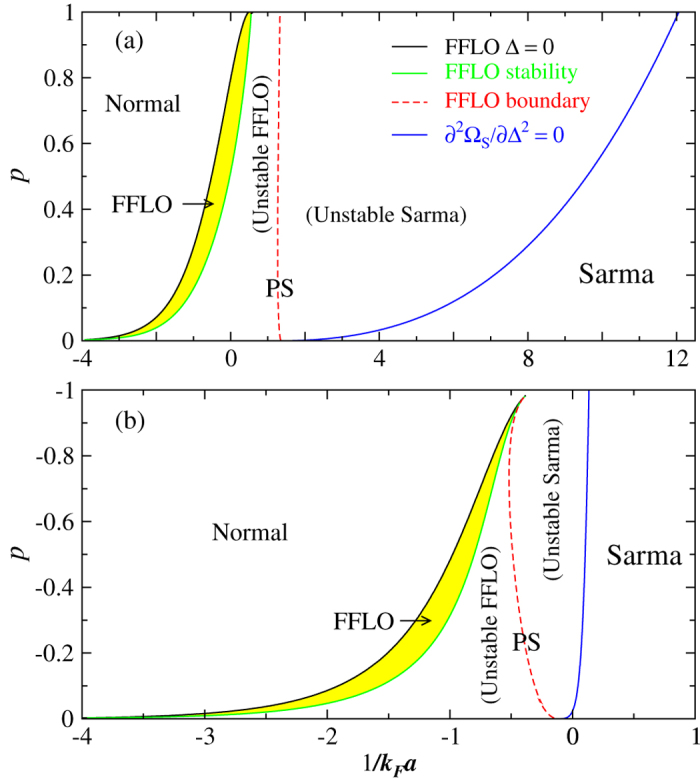
Phase diagram of ^6^Li–^40^K in the *p* − 1/*k*_*F*_*a* plane at *T* = 0 for (**a**) *p* > 0 and (**b**) *p* < 0. Stable FFLO phase lives in the narrow (yellow) shaded regions. Here “PS” labels phase separation (against unstable FFLO and Sarma superfluids), divided by the (red) dashed **q** = 0 line.

Shown in Figs 5 are the calculated *p*–1/*k*_*F*_*a* phase diagrams of a ^6^Li–^40^K mixture at *T* = 0 for (a) *p* > 0 and (b) *p* < 0, respectively. When ^40^K is the majority, Fig. 5(a) shows that a narrow (yellow shaded) region of stable FFLO superfluids persists from the BCS through the near-BEC regime, up to 1/*k*_*F*_*a* ≈ 0.55, as *p* varies from 0 to 1. Apparently, in the near-BEC regime, the stable FFLO phase exists only at large *p*. On the other hand, when ^6^Li is the majority, the stable FFLO phase moves left completely to the BCS side, as shown in Fig. 5(b), in agreement with Figs 1 and 2. In comparison with the equal-mass case^16^, here the stable FFLO region for *p* > 0 is slightly larger, while it becomes smaller for *p* < 0. Here “PS” in both figures labels the regions of FFLO and Sarma superfluids that are unstable against phase separations. In both cases, the FFLO *q* vector increases from 0 in magnitude as |*p*| increases along the boundaries of the stable FFLO phase. The red dashed line separates from unstable FFLO and unstable Sarma regions.

More details about the behavior of *q* as a function of interaction strength and population imbalance *p* are given in the Supplementary Information.

It is interesting to note that for *p* < 0, due to the left shift of the PS phase, the stable zero *T* Sarma superfluid phase has extended into the unitary regime (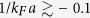) for small |*p*|, as can also be seen in Fig. 1, where the Sarma phase extends all the way down to *T* = 0 at 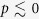. This should be contrasted with the *p* > 0 case and the equal mass case, where zero *T* Sarma superfluid can be found only when 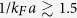 and 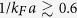, respectively^52^.

By performing vertical and horizontal scans in Fig. 5, we have also studied the behavior of the order parameter at zero *T* as a function of 1/*k*_*F*_*a* and *p*. Typical results are presented in Supplementary Fig. S5. We have found that, as the transition boundary between the FFLO and normal gas phase is approached from within the FFLO phase, the order parameter vanishes as a square root of the distance to the critical point. Such a square root scaling behavior is characteristic of a mean-field theory.

Since a flat bottom or quasi-uniform trap has been realized experimentally^53^, our homogeneous result may be directly applicable when such a trap is used. For a harmonic confining trap which causes inhomogeneity in terms of population imbalances^54^,^55^,^56^, we take the study of the homogeneous case as a necessary first step. In addition, one may obtain experimentally homogeneous result using a tomography technique^27^). In a trap, sandwich-like shell structures will emerge when *p* > 0, with superfluid or pseudogapped normal state in the middle shell^56^,^57^. Figure 1 suggests that the FFLO states may be found locally at low *T* near the shell interfaces where one may find suitable population imbalances.

Finally, we note that while more complex crystalline types of FFLO states are expected to have a slightly lower energy and thus may extend the PS-FFLO phase boundary slightly towards the PS phases in the phase diagrams. While one pair of ±**q** (i.e., the LO state) may lead to a lower energy, higher order crystalline states may further decrease the energy. Nevertheless, the energy difference between the FF and LO states is so small that the phase diagrams calculated using these two different states are almost indistinguishable in the literature^15^,^16^,^17^. Therefore, we expect this to cause only very slight quantitative modifications in the phase diagrams and not to influence our main findings about the enhancement effect of a large mass ratio.

## Conclusions

In summary, our results show that, in order to find the exotic FFLO states in a 3D Fermi gas, it is most promising to explore Fermi-Fermi mixtures with a large mass ratio in the unitary regime, where one expects to see a relatively large phase space volume and a greatly enhanced superfluid transition temperature when the heavy species is the majority. While we have focused on the one-plane-wave FFLO, i.e., the FF case, such enhancement is present for the LO phase as well, which has only a slightly lower energy. These FFLO states may be detected via collective modes^58^, vortices^59^, direct imaging^60^, rf spectroscopy^61^, triplet pair correlations^62^, and, most directly, by measuring the pair momentum distribution which should exhibit a peak at a finite **q**. Experimentally, the 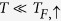 regime has now been accessible for ^6^Li–^40^K^27^. With the recent report of 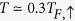
48, it is hopeful that lower *T* regime can be accessed for ^6^Li–^173^Yb as well in the near future. These experimental progress makes it promising to observe the exotic FFLO states if they do exist.

## Additional Information

**How to cite this article:** Wang, J. *et al*. Enhancement effect of mass imbalance on Fulde-Ferrell-Larkin-Ovchinnikov type of pairing in Fermi-Fermi mixtures of ultracold quantum gases. *Sci. Rep.* **7**, 39783; doi: 10.1038/srep39783 (2017).

**Publisher's note:** Springer Nature remains neutral with regard to jurisdictional claims in published maps and institutional affiliations.

## Supplementary Material

Supplementary Information

## References

[b1] Chen Q. J., Stajic J., Tan S. N. & Levin K. BCS-BEC crossover: From high temperature superconductors to ultracold superfluids. Phys. Rep. 412, 1–88 (2005).

[b2] Bloch I., Dalibard J. & Zwerger W. Many-body physics with ultracold gases. Rev. Mod. Phys. 80, 885–964 (2008).

[b3] Fulde P. & Ferrell R. A. Superconductivity in a strong spin-exchange field. Phys. Rev. 135, A550–A563 (1964).

[b4] Larkin A. I. & Ovchinnikov Y. N. Inhomogeneous state of superconductors. Sov. Phys. JETP 20, 762–769 (1965). [Zh. Eksp. Teor. Fiz. **47**, 1136 (1964)].

[b5] Casalbuoni R. & Nardulli G. Inhomogeneous superconductivity in condensed matter and qcd. Rev. Mod. Phys. 76, 263–320 (2004).

[b6] Bianchi A. . First order superconducting phase transition in CeCoIn_5_. Phys. Rev. Lett. 89, 137002 (2002). 12225052 10.1103/PhysRevLett.89.137002

[b7] Lebed A. G. & Wu S. Larkin-Ovchinnikov-Fulde-Ferrell phase in the superconductor (TMTSF)^2^ClO_4_: Theory versus experiment. Phys. Rev. B 82, 172504 (2010).

[b8] Vorontsov A. B., Sauls J. A. & Graf M. J. Phase diagram and spectroscopy of FFLO states of two-dimensional *d*-wave superconductors. Phys. Rev. B 72, 184501 (2005).

[b9] Berridge A. M., Green A. G., Grigera S. A. & Simons B. D. Inhomogeneous magnetic phases: a LOFF-like phase in Sr^3^Ru^2^O_7_. Phys. Rev. Lett. 102, 136404 (2009). 19392379 10.1103/PhysRevLett.102.136404

[b10] Cho K. . Anisotropic upper critical field and a possible Fulde-Ferrel-Larkin-Ovchinnikov state in a stoichiometric pnictide superconductor LiFeAs. Phys. Rev. B 83, 060502R (2011).

[b11] Müther H. & Sedrakian A. Phases of asymmetric nuclear matter with broken space symmetries. Phys. Rev. C 67, 015802 (2003).

[b12] Alford M., Bowers J. & Rajagopal K. Crystalline color superconductivity. Phys. Rev. D 63, 074016 (2001).

[b13] Sedrakian A., Mur-Petit J., Polls A. & Müther H. Pairing in a two-component ultracold Fermi gas: phases with broken space symmetries. Phys. Rev. A 72, 013613 (2005).

[b14] Liao Y.-A. . Spin-imbalance in a one-dimensional Fermi gas. Nature 467, 567–569 (2010). 20882011 10.1038/nature09393

[b15] Radzihovsky L. & Sheehy D. E. Imbalanced Feshbach-resonant Fermi gases. Rep. Prog. Phys. 73, 076501 (2010).

[b16] He Y., Chien C.-C., Chen Q. J. & Levin K. Single-plane-wave Larkin-Ovchinnikov-Fulde-Ferrell state in BCS-BEC crossover. Phys. Rev. A 75, 021602 (2007).

[b17] Sheehy D. E. & Radzihovsky L. BEC-BCS crossover in “magnetized” Feshbach-resonantly paired superfluids. Phys. Rev. Lett. 96, 060401 (2006). 16605966 10.1103/PhysRevLett.96.060401

[b18] Hu H. & Liu X.-J. Mean-field phase diagrams of imbalanced Fermi gases near a Feshbach resonance. Phys. Rev. A 73, 051603 (2006).

[b19] He L., Jin M. & Zhuang P. Finite-temperature phase diagram of a two-component Fermi gas with density imbalance. Phys. Rev. B 74, 214516 (2006).

[b20] Combescot R. & Mora C. Transition to the Fulde-Ferrel-Larkin-Ovchinnikov planar phase : a quasiclassical investigation with Fourier expansion. Phys. Rev. B 71, 144517 (2005).

[b21] Yoshida N. & Yip S.-K. Larkin-Ovchinnikov state in resonant Fermi gas. Phys. Rev. A 75, 063601 (2007).

[b22] Machida K., Mizushima T. & Ichioka M. Generic phase diagram of fermion superfluids with population imbalance. Phys. Rev. Lett. 97, 120407 (2006). 17025946 10.1103/PhysRevLett.97.120407

[b23] Zhang W. & Duan L.-M. Finite-temperature phase diagram of trapped Fermi gases with population imbalance. Phys. Rev. A 76, 042710 (2007).

[b24] Kinnunen J., Jensen L. M. & Törmä P. Strongly interacting Fermi gases with density imbalance. Phys. Rev. Lett. 96, 110403 (2006). 16605801 10.1103/PhysRevLett.96.110403

[b25] Zwierlein M. W., Schirotzek A., Schunck C. H. & Ketterle W. Fermionic superfluidity with imbalanced spin populations. Science 311, 492 (2006). 16373535 10.1126/science.1122318

[b26] Partridge G. B., Li W., Kamar R. I., Liao Y. A. & Hulet R. G. Pairing and phase separation in a polarized Fermi gas. Science 311, 503 (2006). 16373534 10.1126/science.1122876

[b27] Shin Y.-I., Schunck C. H., Schirotzek A. & Ketterle W. Phase diagram of a two-component Fermi gas with resonant interactions. Nature 451, 689–693 (2008). 18256666 10.1038/nature06473

[b28] Gubbels K. B., Baarsma J. E. & Stoof H. T. C. Lifshitz point in the phase diagram of resonantly interacting ^6^Li–^40^K mixtures. Phys. Rev. Lett. 103, 195301 (2009). 20365934 10.1103/PhysRevLett.103.195301

[b29] Baarsma J. E., Gubbels K. B. & Stoof H. T. C. Population and mass imbalance in atomic Fermi gases. Phys. Rev. A 82, 013624 (2010).

[b30] Baarsma J. E. & Stoof H. T. C. Inhomogeneous superfluid phases in ^6^Li-^40^K mixtures at unitarity. Phys. Rev. A 87, 063612 (2013).

[b31] Zheng Z., Gong M., Zou X., Zhang C. & Guo G. Route to observable Fulde-Ferrell-Larkin-Ovchinnikov phases in three-dimensional spin-orbit-coupled degenerate Fermi gases. Phys. Rev. A 87, 031602 (2013).

[b32] Liu X.-J. & Hu H. Inhomogeneous Fulde-Ferrell superfluidity in spin-orbit-coupled atomic Fermi gases. Phys. Rev. A 87, 051608(R) (2013). 10.1103/PhysRevLett.113.115302 25259986

[b33] Zhou X.-F., Guo G.-C., Zhang W. & Yi W. Exotic pairing states in a Fermi gas with three-dimensional spin-orbit coupling. Phys. Rev. A 87, 063606 (2013).

[b34] Wu F., Guo G.-C., Zhang W. & Yi W. Unconventional superfluid in a two-dimensional Fermi gas with anisotropic spin-orbit coupling and Zeeman fields. Phys. Rev. Lett. 110, 110401 (2013). 25166515 10.1103/PhysRevLett.110.110401

[b35] Dong L., Jiang L. & Pu H. Fulde-Ferrell pairing instability in spin-orbit coupled Fermi gas. New J. Phys. 15, 075014 (2013).

[b36] Hu H. & Liu X.-J. Fulde-Ferrell superfluidity in ultracold Fermi gases with Rashba spin-orbit coupling. New J. Phys. 15, 093037 (2013).

[b37] Iskin M. Spin-orbit coupling induced Fulde-Ferrell-Larkin-Ovchinnikov-like Cooper pairing and skyrmion-like polarization textures in trapped optical lattices. Phys. Rev. A 88, 013631 (2013).

[b38] Cai Z., Wang Y. & Wu C. Stable Fulde-Ferrell-Larkin-Ovchinnikov pairing states in 2D and 3D optical lattices. Phys. Rev. A 83, 063621 (2011).

[b39] Franca V. V., Hördlein D. & Buchleitner A. Fulde-ferrell-larkin-ovchinnikov critical polarization in one-dimensional fermionic optical lattices. Phys. Rev. A 86, 033622 (2012).

[b40] Mendoza R., Fortes M., Solís M. A. & Koinov Z. Superfluidity of a spin-imbalanced Fermi gas in a three-dimensional optical lattice. Phys. Rev. A 88, 033606 (2013).

[b41] Okawauchi Y. & Koga A. Stability of FFLO states in optical lattices with bilayer structure. J. Phys. Soc. Jpn. 81, 074001 (2012).

[b42] Kim D.-H. & Törmä P. Fulde-ferrell-larkin-ovchinnikov state in the dimensional crossover between one- and three-dimensional lattices. Phys. Rev. B 85, 180508(R) (2012).

[b43] Chen A.-H. & Gao X. L. Pure Fulde-Ferrell-Larkin-Ovchinnikov state in optical lattices. Phys. Rev. B 85, 134203 (2012).

[b44] Wille E. . Exploring an ultracold Fermi-Fermi mixture: Interspecies Feshbach resonances and scattering properties of ^6^Li and ^40^K. Phys. Rev. Lett. 100, 053201 (2008). 18352370 10.1103/PhysRevLett.100.053201

[b45] Naik D. . Feshbach resonances in the ^6^Li–^40^K Fermi-Fermi mixture: elastic versus inelastic interactions. Eur. Phys. J. D 65, 55–65 (2011).

[b46] Taglieber M., Voigt A.-C., Aoki T., Hänsch T. W. & Dieckmann K. Quantum degenerate two-species Fermi-Fermi mixture coexisting with a Bose-Einstein condensate. Phys. Rev. Lett. 100, 010401 (2008). 18232744 10.1103/PhysRevLett.100.010401

[b47] Fukuhara T., Takasu Y., Kumakura M. & Takahashi Y. Degenerate Fermi gases of ytterbium. Phys. Rev. Lett. 98, 030401 (2007). 17358662 10.1103/PhysRevLett.98.030401

[b48] Hansen A. H. . Production of quantum-degenerate mixtures of ytterbium and lithium with controllable interspecies overlap. Phys. Rev. A 87, 013615 (2013).

[b49] Pao C.-H., Wu S.-T. & Yip S.-K. Superfluid stability in the BEC-BCS crossover. Phys. Rev. B 73, 132506 (2006).

[b50] Chen Q. J., He Y., Chien C.-C. & Levin K. Stability conditions and phase diagrams for two-component Fermi gases with population imbalance. Phys. Rev. A 74, 063603 (2006).

[b51] Guo H., Chien C.-C., Chen Q. J., He Y. & Levin K. Finite-temperature behavior of an interspecies fermionic superfluid with population imbalance. Phys. Rev. A 80, 011601 (2009).

[b52] Chien C. C., Chen Q. J., He Y. & Levin K. Intermediate temperature superfluidity in a Fermi gas with population imbalance. Phys. Rev. Lett. 97, 090402 (2006). 17026346 10.1103/PhysRevLett.97.090402

[b53] Gaunt A. L., Schmidutz T. F., Gotlibovych I., Smith R. P. & Hadzibabic Z. Bose-Einstein condensation of atoms in a uniform potential. Phys. Rev. Lett. 110, 200406 (2013). 25167389 10.1103/PhysRevLett.110.200406

[b54] Chien C. C., Chen Q. J., He Y. & Levin K. Superfluid phase diagrams of trapped Fermi gases with population imbalance. Phys. Rev. Lett. 98, 110404 (2007). 17501030 10.1103/PhysRevLett.98.110404

[b55] Pao C.-H., Wu S.-T. & Yip S.-K. Asymmetric Fermi superfluid with different atomic species in a harmonic trap. Phys. Rev. A 76, 053621 (2007).

[b56] Wang J. B., Guo H. & Chen Q. J. Exotic phase separation and phase diagrams of a Fermi-Fermi mixture in a trap at finite temperature. Phys. Rev. A 87, 041601 (2013).

[b57] Chen Q. J. Effect of the particle-hole channel on BCS-Bose-Einstein condensation crossover in atomic Fermi gases. Sci. Rep. 6, 25772 (2016). 27183875 10.1038/srep25772 PMC4868972

[b58] Edge J. M. & Cooper N. Signature of the FFLO phase in the collective modes of a trapped ultracold Fermi gas. Phys. Rev. Lett. 103, 065301 (2009). 19792579 10.1103/PhysRevLett.103.065301

[b59] Agterberg D., Zheng Z. & Mukherjee S. Spatial line nodes and fractional vortex pairs in the Fulde-Ferrell-Larkin-Ovchinnikov phase. Phys. Rev. Lett. 100, 017001 (2008). 18232806 10.1103/PhysRevLett.100.017001

[b60] Mizushima T., Machida K. & Ichioka M. Direct imaging of spatially modulated superfluid phases in atomic fermion systems. Phys. Rev. Lett. 94, 060404 (2005). 15783711 10.1103/PhysRevLett.94.060404

[b61] Bakhtiari M. R., Leskinen M. J. & Torma P. Spectral signatures of the Fulde-Ferrell-Larkin-Ovchinnikov order parameter in one-dimensional optical lattices. Phys. Rev. Lett. 101, 120404 (2008). 18851346 10.1103/PhysRevLett.101.120404

[b62] Zapata I., Sols F. & Demler E. Triplet pair correlations in *s-*wave superfluids as a signature of the FFLO state. Phys. Rev. Lett. 109, 155304 (2012). 23102326 10.1103/PhysRevLett.109.155304

