# Supplementary material for: Enhancement effect of mass imbalance on Fulde-Ferrell-Larkin-Ovchinnikov type of pairing in Fermi-Fermi mixtures of ultracold quantum gases
Source: Sci Rep. 2017 Jan 4;7:39783. doi: 10.1038/srep39783 (PMC5209654; doi:10.1038/srep39783)
Supplement: Supplementary Information [file srep39783-s1.pdf]

Supplementary Information

Enhancement effect of mass imbalance on Fulde-Ferrell-Larkin-Ovchinnikov type of pairing in Fermi-Fermi mixtures of ultracold quantum gases

Jibiao Wang, Yanming Che, Leifeng Zhang, and Qijin Chen*

Department of Physics and Zhejiang Institute of Modern Physics, Zhejiang University, Hangzhou, Zhejiang 310027, China and Synergetic Innovation Center of Quantum Information and Quantum Physics, Hefei, Anhui 230026, China

*Email: qchen@zju.edu.cn

(Dated: November 5, 2016)

Abstract

Here we present extra, supplemental information for the main text. We show how the enhancement effect of mass imbalance on the FFLO type of pairing may appear different when the phase diagram is plotted using different definitions of the Fermi energy as the energy unit. Also shown are the behavior of the FFLO momentum $\mathbf{q}$ as a function of interaction strength and population imbalance and the behavior of the order parameter amplitude as a function of pairing strength and population imbalance.

I. EFFECT OF LARGE MASS RATIO: EVOLUTION OF THE FFLO PHASE WITH TEMPERATURE MEASURED IN DIFFERENT UNITS

As discussed in the main text, the evolution of the FFLO transition temperature as a function of T/T_F has to do with the definition of T_F. We have used the average mass m and balanced k_F to define T_F in the main text. Here we plot the evolution of the FFLO phase using a different definition of Fermi temperature as our energy unit.

First, we use $T_{F\uparrow}$, the Fermi temperature of the heavy atoms, as the energy unit. This is the choice of the experimental work of Shin et al [Nature 451, 689 (2008)]. The result is shown in Fig. S1. In comparison with Fig. 4 in the main text, here we obtain a much greater enhancement of the transition temperature. In comparison with the equal mass case (the black curve, labeled 1:1), for the mixtures of ^{6}Li–^{40}K and ^{6}Li–^{173}Yb, T_c is enhanced by a factor of about 7 and 16, respectively.

Next, we replot the same figure using a different Fermi temperature definition, $T_{F,r}$, as the energy unit. Here $T_{F,r} \equiv k_F^2/2m_r$, where $m_r = 2m_\uparrow m_\downarrow/(m_\uparrow + m_\downarrow)$ is defined in the main text. The result is presented in Fig. S2. In this new plot, the enhancement of T_c by mass imbalance is far less dramatic. This essentially exhibits the least enhancement effect among all different

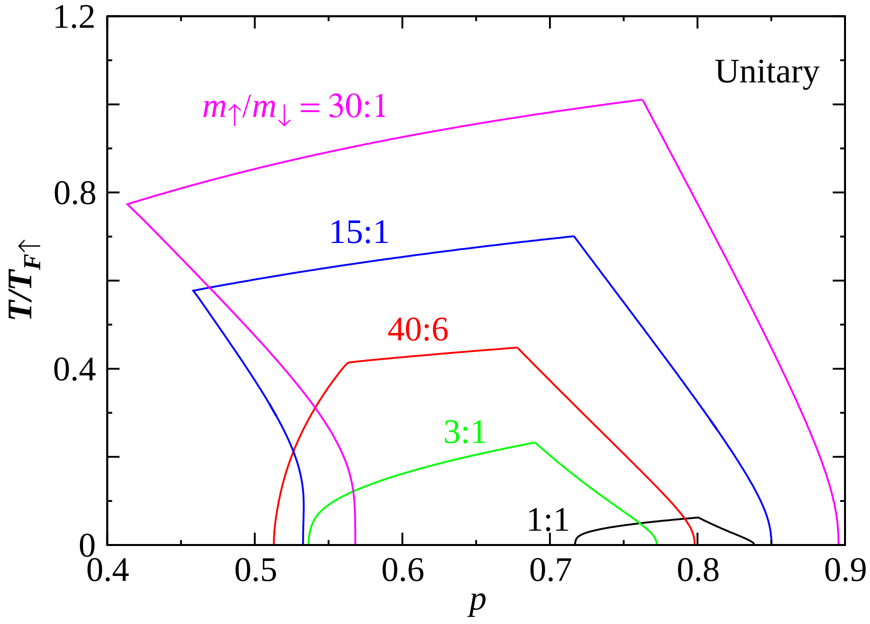

Figure S1. (Color) T–p phase diagram of stable FFLO superfluid in Fermi-Fermi mixtures with different mass ratios (as labeled) at unitarity. A large mass ratio enhances FFLO type of pairing. Here the temperature is measured in units of $T_{F\uparrow}$.

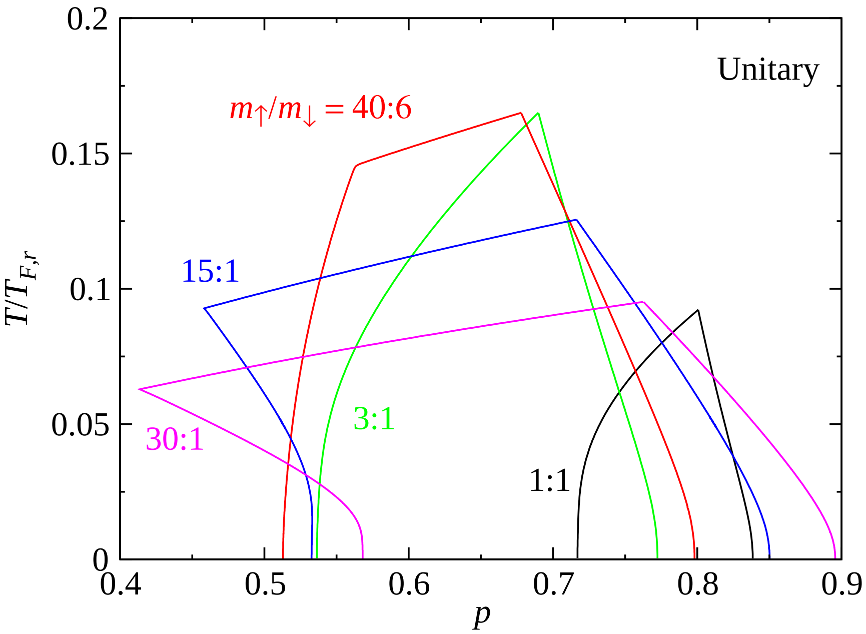

Figure S2. (Color) T–p phase diagram of stable FFLO superfluid, same as Fig. S1, except that here temperature is measured in (inappropriate) units of $T_{F,r}$. The enhancement of T_c does not exhibit an monotonic order when plotted this way.

choices of T_F. However, as discussed in the main text, $T_{F,r}$ is not an appropriate definition since m_r puts erroneously more weight on the light species, while we are focusing on the heavy species dominant regime.

In all cases, however, in terms of the phase space area, the enhancement of a large mass ratio $m_\uparrow/m_\downarrow$ is dramatic.

II. EVOLUTION OF THE FFLO WAVEVECTOR q

In this section, we present the result of the FFLO wave vector $\mathbf{q}$ at zero T as a function of $1/k_Fa$ from BCS to BEC and population imbalance p, for the case of ^{6}Li–^{40}K mixture. The result is far from trivial.

First, we replot Fig. 4(b) for $p < 0$ from the main text in Fig. S3(a) but with more details. The red dashed line is where the LOFF wave vector $\mathbf{q}$ drops to zero from the left. Interestingly, as shown in Fig. S3(c), the vector $\mathbf{q}$ does not drop to zero in a simple monotonic way. Instead, for a given p, as a function of $1/k_Fa$, it first increases to a maximum, then decreases, and then bends back, followed by a quick drop to zero. The trace of the back-bending point, determined via $\partial(1/k_Fa)/\partial q = 0$, is plotted as the magenta dot-dashed curve. This is the right most boundary for

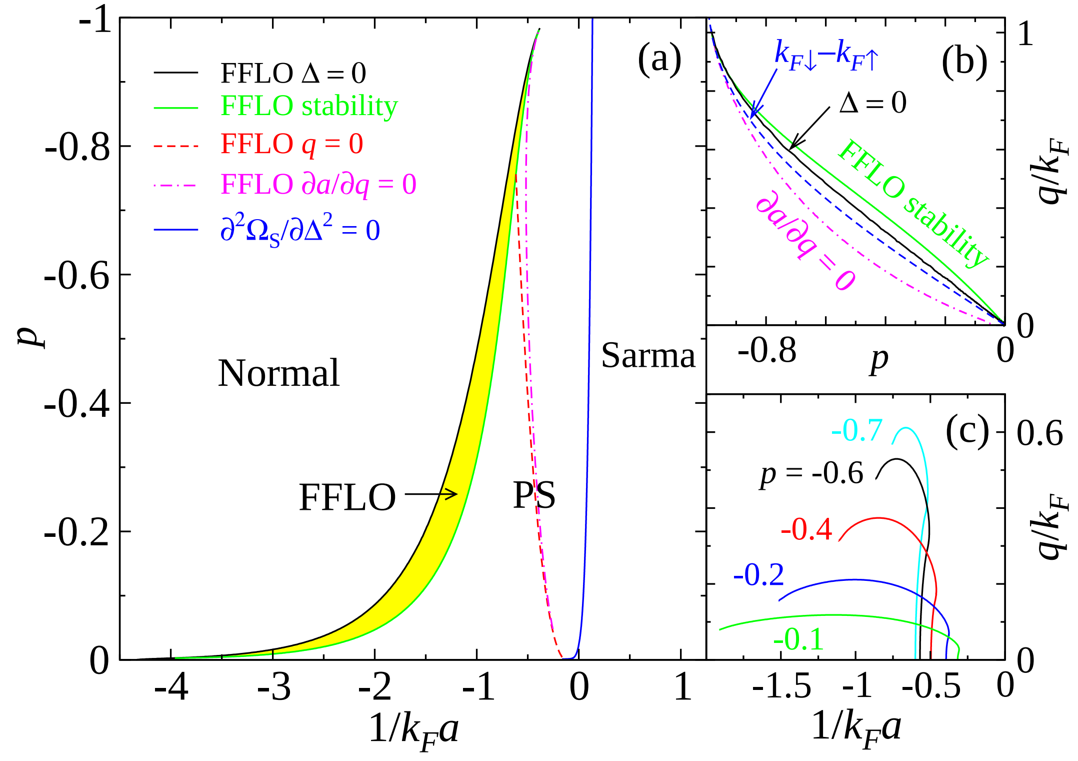

Figure S3. (a) Zero temperature phase diagram in the p–$1/k_Fa$ plane for $p < 0$, where the ^{6}Li is the majority. (b) Momentum q/k_F as a function of p along the boundaries of the FFLO phase as well as the $\partial a/\partial p = 0$ line. For comparison, also plotted is δk_F. (c) Momentum q/k_F as a function of $1/k_Fa$ for a series of imbalance p, as labeled.

nonzero q, and is represented by the red dashed line in Fig. 4(b) in the main text. This means that in the narrow region between the red $q = 0$ line and the magenta $\partial a/\partial q = 0$ line, the momentum q is double valued; there exist two solutions of FFLO states with different wavevectors for the system. Similar back-bending behavior of q versus $1/k_Fa$ has been seen in the mass balanced case [Y. He, C.-C. Chien, Q. J. Chen and K. Levin, Phys. Rev. A **75**, 021602(R) (2007)].

Figure S3(c) reveals that on the left edge of the FFLO phase, where the gap Δ drops to zero, the momentum q is nonzero. In Fig. S3(b), we plot q/k_F along three different lines as a function of p, namely, the (black) $\Delta = 0$ boundary, the (green) FFLO stability boundary, and the (magenta) back-bending line. Along all three lines, q increases with imbalance $|p|$, as one may expect from the increasing mismatch between the Fermi surfaces.

For comparison, we also plot the Fermi momentum difference $\delta k_F = |k_{F\uparrow} - k_{F\downarrow}|$ (blue dashed line) in Fig. S3(b). To the zeroth order approximation, q is given by this difference.

The back-bending behavior of q versus $1/k_Fa$ does not occur for the case of $p > 0$, where the heavy atoms dominate. Momentum q drops to zero at the red dashed line in Fig. 5(a) in the main

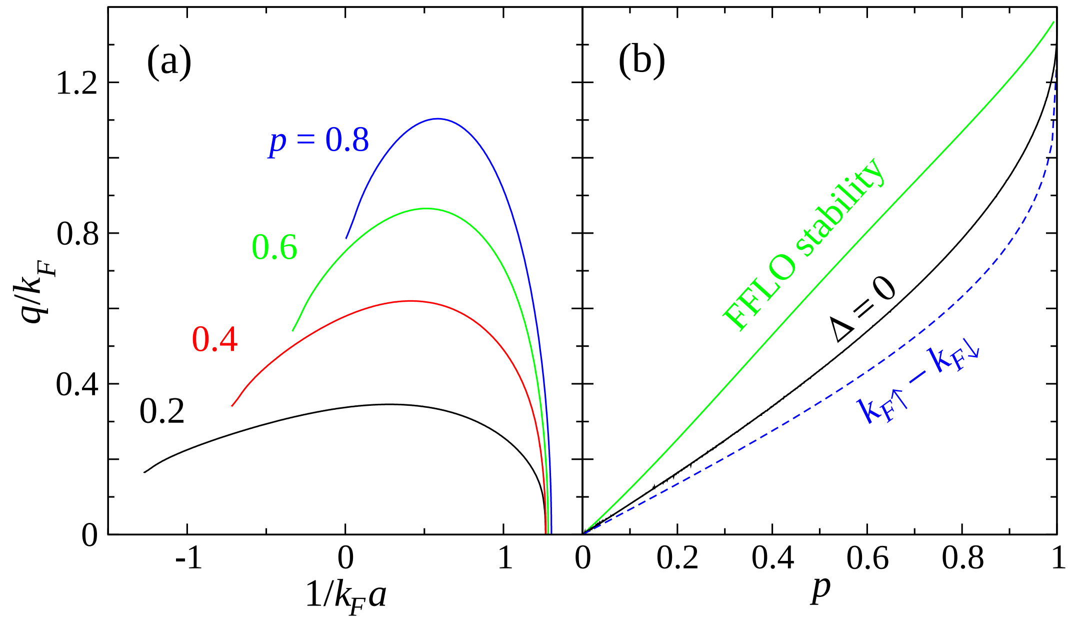

Figure S4. (a) Momentum q as a function of $1/k_F a$ for a series of imbalance p, as labeled, at zero T, when ^{40}K is the majority. (b) Momentum q as a function of p along the boundaries of the FFLO phase. The blue dashed line shows δk_F. See Fig. 4(a) in the main text for the phase diagram.

text. Similarly, we plot in Fig. S4(a) q/k_F as a function of $1/k_F a$ for different imbalances p at zero T. The behavior of q versus $1/k_F a$ looks more regular, even though it still exhibits a monotonic hump behavior for a given p. As shown in Fig. S4(b), along the boundaries of the FFLO phase, q increases with p as well. While the value of q along the (black) $\Delta = 0$ boundary is roughly given by δk_F (blue dashed line), it shows a big deviation along the (green) FFLO stability line. When compared with the $p < 0$ case, we notice that here the value of q is slightly larger.

III. BEHAVIORS OF THE ORDER PARAMETER

In the main text, we have shown the typical behavior of the order parameter amplitude Δ in the FFLO phase as a function of T for both unitary and BCS cases. Here we show more results regarding the behavior of Δ.

Shown in Fig. S5 is the zero temperature gap Δ as a function of (a) population imbalance p at unitarity and (b) of $1/k_F a$ with $p = 0.5129$, for a homogeneous ^{6}Li-^{40}K mixture. The plots for the BCS case are very similar. Fig. S5(a) corresponds to a vertical scan in Fig. 5(a) of the main text along the $1/k_F a = 0$ line, which intersects the FFLO-PS phase boundary at $p = 0.5129$. Therefore, the curve in Fig. S5(a) starts at $p = 0.5129$ and ends near $p = 0.8$, where the FFLO

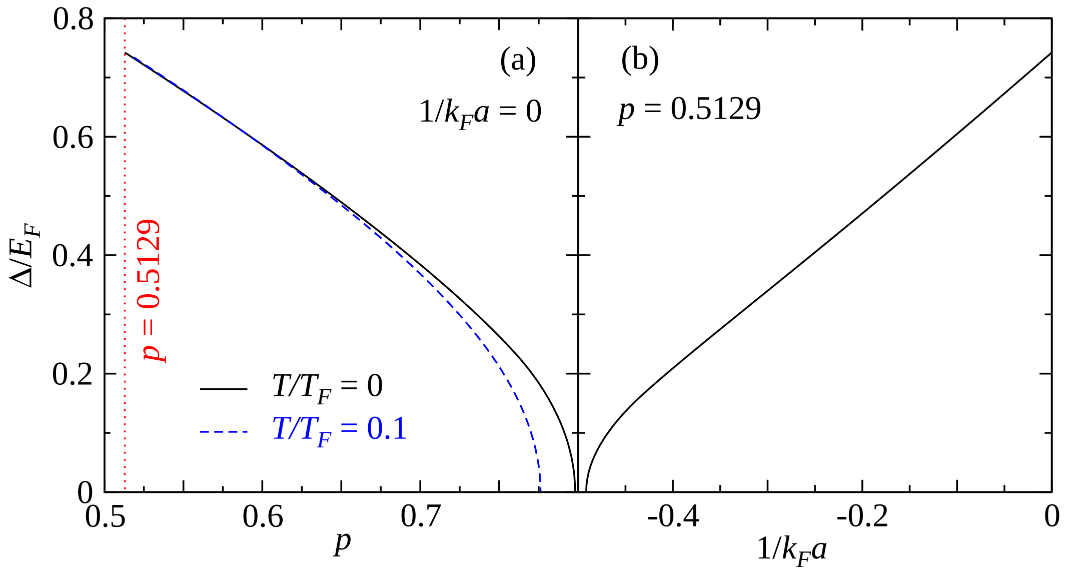

Figure S5. Behavior of the zero T gap Δ (a) at unitarity as function of imbalance p and (b) at $p = 0.5129$ as a function of $1/k_F a$, for a homogeneous ^{6}Li-^{40}K mixture.

phase gives way to the normal state. The gap Δ decreases with p and vanishes when it hits the FFLO-normal phase boundary. We also show a similar scan but at $T/T_F = 0.1$, as the blue dashed line. Except for a slightly different range, it is very close to the zero T curve. Fig. S5(b) corresponds to a horizontal scan in Fig. 5(a) along the $p = 0.5129$ line. The gap increases from zero at the FFLO-normal phase boundary on the BCS side and reaches the maximum at unitarity, where the FFLO-PS phase transition takes place. For both Figs. S5(a) and (b), the gap vanishes with a square root power law towards the FFLO-normal phase boundary, i.e., $\Delta \propto \sqrt{\delta}$, where δ measures the distance away from the FFLO-normal phase transition point. The scaling behavior with a critical exponent $1/2$ is characteristic of a mean-field theory.

In Fig. S6, we plot the ratio $2\Delta_0/T_c$ for the FFLO states of a homogeneous ^{6}Li-^{40}K mixture at unitarity, as a function of population imbalance p. Here Δ_0 is the zero T gap, and p is restricted the values at which the FFLO-normal gas transition exists at T_c. This is the range of p spanned by the FFLO-normal phase boundary in Fig. 1 of the main text. Fig. S6 suggests that the ratio varies with a rather big range, and can be either bigger or smaller than the value 3.52 for a conventional homogeneous BCS superfluid in three dimensions.

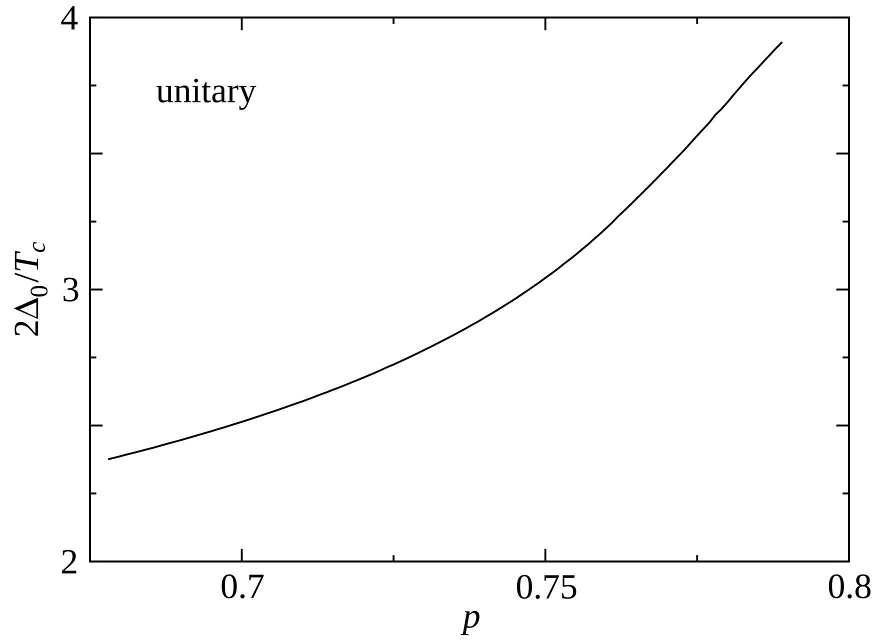

Figure S6. Behavior of the ratio of $2\Delta_0/T_c$ for a homogeneous ^{6}Li-^{40}K mixture at unitarity, for population imbalances such that the FFLO-normal phase transition exists.
